# Infant Feeding Beliefs, Attitudes, Knowledge and Practices of Chinese Immigrant Mothers: An Integrative Review of the Literature

**DOI:** 10.3390/ijerph15010021

**Published:** 2017-12-23

**Authors:** Ana Cristina Lindsay, Qun Le, Mary L. Greaney

**Affiliations:** 1 Department of Exercise and Health Sciences, University of Massachusetts Boston, Boston, MA 02125, USA; Qun.Le001@umb.edu; 2 Department of Nutrition, Harvard T. H. Chan School of Public Health, Boston, MA 02115, USA; 3 Health Studies and Department of Kinesiology, University of Rhode Island, Kingston, RI 02881, USA; mgreaney@uri.edu

**Keywords:** breastfeeding, complementary feeding, Chinese, immigrant mothers, infant, obesity

## Abstract

Chinese are a fast-growing immigrant population group in several parts of the world (e.g., Australia, Canada, Europe, Southeast Asia, United States). Research evidence suggests that compared to non-Hispanic whites, individuals of Asian-origin including Chinese are at higher risk of developing cardiovascular disease and type 2 diabetes at a lower body mass index (BMI). These risks may be possibly due to genetic differences in body composition and metabolic responses. Despite the increasing numbers of Chinese children growing up in immigrant families and the increasing prevalence of obesity among Chinese, little research has been focused on children of Chinese immigrant families. This integrative review synthesizes the evidence on infant feeding beliefs, attitudes, knowledge and practices of Chinese immigrant mothers; highlights limitations of available research; and offers suggestions for future research. Using the Preferred Reporting Items for Systematic Review and Meta-Analyses (PRISMA) guidelines, we searched four electronic academic/research databases (CINAHL, Medline, PsycINFO, and PubMed) to identify peer-reviewed, full-text papers published in English between January 2000 and September 2017. Only studies with mothers 18+ years old of normally developing infants were included. Of the 797 citations identified, 15 full-text papers were retrieved and 11 studies (8 cross-sectional studies, 3 qualitative studies) met the inclusion criteria and were included in this review. Reviewed studies revealed high initiation rates of breastfeeding, but sharp declines in breastfeeding rates by six months of age. In addition, reviewed studies revealed that the concomitantly use of breast milk and formula, and the early introduction of solid foods were common. Finally, reviewed studies identified several familial and socio-cultural influences on infant feeding beliefs and practices that may increase risk of overweight and obesity during infancy and early childhood among Chinese children of immigrant families. Nonetheless, as only 11 studies were identified and because the majority of studies (*n* = 8) were conducted in Australia, additional research including longitudinal studies, and studies conducted in countries with large Chinese immigrant population are needed to further identify and understand influences on Chinese immigrant mothers’ beliefs, attitudes, and practices related to infant feeding that may increase risk of child overweight and obesity. This information is needed to develop interventions tailored to the beliefs and needs of this fast-growing immigrant group and aimed at promoting healthy infant feeding practices to prevent childhood overweight and obesity.

## 1. Introduction

Childhood overweight and obesity are significant global public health problems [1,2] and prior research documents increased risk of overweight and obesity among racial/ethnic minority children of immigrant families [3,4]. More specifically, evidence suggests that children from racial/ethnic minority, immigrant families with low incomes are at elevated risk of obesity in the first two years of life [5,6,7,8,9,10,11]. Lower socio-economic status, limited education language barriers, cultural beliefs, and inadequate access to health care have been found to be associated with increased risk of overweight and obesity among children of immigrant parents [6,7,8,9,10,11,12].

Chinese are a fast-growing immigrant population group in several parts of the world (e.g., Australia, Canada, Europe, Southeast Asia, United States (U.S.)). Research evidence suggests that compared to non-Hispanic whites individuals of Asian-origin including Chinese develop cardiovascular disease and type 2 diabetes at a lower body mass index (BMI). This difference may be due to genetic differences in body composition and metabolic responses [13,14]. Despite the increasing numbers of Chinese children of immigrant families, and the overall increasing prevalence of obesity among Chinese immigrants, until recently children of Chinese immigrants have received little attention in the childhood obesity research literature [15,16,17,18].

Infancy is a crucial developmental period that has long-term impact on children’s health status [19,20,21]. Identifying modifiable risk factors amenable to interventions during this early life stage is critical for preventing childhood obesity and its associated co-morbidities [21,22,23]. Parents, especially mothers, make or directly influence the early feeding decisions and practices that impact their children’s healthy eating habits, food preferences, ability to self-regulate food intake, and ultimately risk of overweight and obesity in early childhood and later life [24,25,26,27,28,29,30]. Evidence suggests that non-exclusive and limited breastfeeding (less than four months) and the early introduction of foods (e.g., serving juices, adding cereal to the bottle) are associated with rapid growth in the first six months of life and increased risk of obesity in infancy and early childhood, which may carry into later life stages [27,28,31,32,33,34,35,36,37,38]. Nonetheless, the worldwide prevalence of exclusive breastfeeding (EBF) is low and early introduction of complementary feeding is high [22].

Several national and international organizations recommend EBF infants until six months of age, then continuing breastfeeding until at least one year of age with nutritious solid foods being introduced at about six months [24,38]. Parents’ decision to breastfeed is one of the first feeding decisions they make and this decision may have a lasting impact on their children’s weight and health status [39]. Results of research examining the effects of breastfeeding on risk of child obesity are not consistent, with some research indicating that breastfeeding protects against overweight and obesity during childhood [33,39]. Results of some studies, however, indicate that breastfeeding has minimal [40] or no impact [41] on children’s weight status.

Complementary feeding, the process of gradual introduction of foods and beverages to breastfed or formula-fed infants, is an important feeding transition that has been linked to eating habits, food preferences and risk of excessive weight gain and obesity in children [28,30,33,34]. Research evidence suggests that introduction of complementary foods before four months of age (risk may be even stronger among formula-fed infants) is a risk factor for obesity [42,43].

It is important to identify factors associated with infant feeding beliefs, attitudes and practices that may inhibit children developing healthy eating habits and weight so interventions to prevent and reduce child obesity can be developed. Interventions that offer parents guidance on healthy infant feeding practices may be an important strategy to promote children’s healthy weight status [23,24,25,26]. Given that Chinese are at increased risk of cardiovascular disease and type 2 diabetes at a lower BMI [13,14,15,16,17,18], and the growing evidence linking early feeding practices to the risk of childhood obesity [44,45], the purpose of this integrative literature review were to: (1) identify and summarize findings from existing studies examining infant feeding beliefs, attitudes, knowledge and practices of Chinese-born immigrant mothers; (2) highlight the limitations of reviewed studies; and (3) generate suggestions for future research.

## 2. Materials and Methods

The methods employed during this review were informed by those developed by Whittemore and Knafl [46] and allowed for the inclusion of qualitative, quantitative, and mixed methods studies. The review included three key steps: (1) a systematic literature search; (2) data evaluation involving a thematic analysis process—data reduction, data display, drawing and verifying conclusions; and (3) presentation of conclusions. In addition, we used the reporting guidelines of the Preferred Reporting Items for Systematic Reviews and Meta-Analysis (PRISMA) statement [47] to guide the inclusion and exclusion of research papers. The PRISMA statement comprises guidelines that include a four-phase flow diagram to systematically guide the inclusion and exclusion of research papers. In addition, the guidelines provide a 27-item checklist that describes the requirements per review section (e.g., title, abstract, introduction, methods, results, discussions, funding) to ensure that systematic reviews are properly conducted and reported [47].

### 2.1. Search Strategy

We searched four electronic databases—PubMed, Medline, PsycINFO, and Cumulative Index to Nursing and Allied Health Literature (CINAHL). The search, conducted between December 2016 and September 2017, was limited to full-text, peer-reviewed articles published in English between January 2000 and September 2017. Search terms included: (1) infan* OR newborns OR child*; (2) ‘breastfeeding’ OR ‘breast milk feeding’ OR ‘breast milk’ OR ‘human milk’ OR ‘nursing’ OR ‘lactation’; (3) ‘formula feeding’ OR formula; (4) ‘complementary feeding’ OR ‘complementary food’ OR ‘supplementary food’; (5) weaning; (6) ‘infant feeding’; (7) ‘feeding practices’ OR ‘feeding behavior’ OR ‘feeding strategy’; (8) parent* OR caregiver OR mother; (9) immigrant; and (10) Chinese OR China.

Two authors (ACL, QL) independently examined the titles and abstracts of all identified citations and studies were excluded when both authors determined that the study did not meet the inclusion criteria. Next, these same two authors independently reviewed the full article of studies that were not excluded based on titles or abstracts. Furthermore, to identify additional potentially eligible studies, these two authors searched the reference lists of existing full articles that satisfied the inclusion criteria. The two authors agreed upon a final set of articles and examined the articles to extract the relevant information pertaining to the objectives of this review. The search strategy using the PRISMA flow diagram is illustrated in Figure S1.

### 2.2. Study Selection

This review was limited to studies that included normally developing children (i.e., not born preterm, not diagnosed with physical or mental complications, etc.) of Chinese immigrants. Qualitative and quantitative studies were eligible for inclusion if they met the following criteria: (1) peer-reviewed, full-text articles published in English between January 2000 and June 2017; (2) sample included Chinese immigrant parents (18+ years old); and (3) in the case of multi-ethnic samples, at least 25% of the total sample was Chinese-born immigrant mothers.

### 2.3. Data extraction and Data Synthesis

The 11 identified eligible studies were analyzed and synthesized using the Matrix Method [48]. Two authors (ACL, QL) independently read all articles and completed a data extraction form created to gather the following: (1) authors; (2) study setting; (3) study aim(s); (4) study population; (5) study design; (6) measure(s) of infant feeding practices; and (7) study findings. The two sets of completed data extraction forms were compared, and discrepancies were resolved with feedback from a third author. Due to the inclusion of studies using qualitative and quantitative designs conducting a meta-analysis of the data was not appropriate, and results of this review are presented as a narrative summary.

### 2.4. Quality Assessment of Included Studies

Included studies were evaluated using two quality frameworks. Using the Strengthening in the Reporting of Observational Studies in Epidemiology (STROBE) guidelines [49], two authors assessed observational studies (*n* = 8) for possible bias and methodological areas that may have been inadequately addressed using a quality checklist created for this review. The checklist consisted of nine questions (see Table S1) designed to be answered with either “yes”, or “no.” Each “yes” response was assigned 1 point, and a score of 0 was given to “no” response). Total scores (range of scores 0–9) were then used to assign a rating of the study as strong (score > 7), moderate (score between 7 and 5), or weak (score < 5). Qualitative studies were assessed using the Critical Appraisal Skills Program (CASP) [50], a nine-question appraisal tool (see Table S2). Two researchers (ACL, QL) independently assessed the studies included in this review using these checklists and discussed and resolved any differences in scoring.

## 3. Results

### 3.1. Search

The search strategy generated 797 unique articles (see Figure S1). After exclusion, 11 research articles were deemed eligible and included in this integrative review [51,52,53,54,55,56,57,58,59,60,61].

### 3.2. Summary of Included Studies

Of the 11 included studies, all focused on breastfeeding and/or formula feeding beliefs, attitudes, knowledge and practices (initiation and duration) [51,52,53,54,55,56,57,58,59,60,61], while five also examined complementary feeding beliefs and practices [54,56,57,60,61]. The 11 studies took place in four countries—eight in Australia, one in Canada, one Ireland, and one in the United States. Of the 11 reviewed studies, eight employed quantitative methods, and all were cross-sectional [51,52,53,54,55,57,58,59]. Sample sizes ranged from 15 to 506. Three quantitative studies (3/8) [51,52,57] used questionnaires specifically developed for the study, either alone or in combination with medical records [52], while three (3/8) [53,54,55] used the Perth Infant Feeding Study (PIFS) questionnaire [62], and two (2/8) [58,59] used the Iowa Infant Feeding Attitudes Survey (IIFAS) [63] translated and adapted for Chinese.

Three studies employed qualitative methods [56,60,61], and all three used semi-structured in-depth interviews. Further details of included studies are presented in Table S3, while Table S4 provides the synthesized information on methodology and main findings of included studies.

#### 3.2.1. Initiation and Duration of Breastfeeding

Seven studies, all conducted in Australia, examined initiation and duration of breastfeeding [51,52,53,54,55,58,59]. These studies found high breastfeeding initiation rates (53.2%–94.1%) among Chinese immigrant mothers, but lower rates of breastfeeding duration in comparison to initiation rates. For example, one study conducted in Australia [51] showed that only 34% of mothers were breastfeeding when baby was three months of age. Similarly, two other studies conducted in Australia [54,55,58] showed sharp decline in breastfeeding (any breastfeeding) rates at six months of age—6% [55] and 55.6% [54]. Similarly, another study conducted in Australia showed that only 33.8% of Chinese immigrant mothers reported “fully breastfeeding” at six months of age [58].

Several studies indicated that most mothers do not EBF for the first six months of life, and use formula concomitantly with breast milk [52,53,54,55,58,59]. One cross-sectional study [55] conducted in Australia found that 88.5% of women initiated breastfeeding; however, only 55.6% reported breastfeeding six-month postpartum. The majority of studies reported that the mean duration of exclusive breastfeeding was below the recommended about six months, with mean duration ranging from two to five months postpartum. Moreover, one study [54] found that duration of breastfeeding was associated with the age at which the infant was introduced to formula or cow’s milk, with a later the introduction being associated with a longer the duration of breastfeeding.

Two cross-sectional studies using data from the same sample [53,54] showed that the most often cited reason for bottle-feeding was a perceived lack of breast milk. Another common reason for stopping breastfeeding reported was mother going back to work [53,54].

#### 3.2.2. Beliefs, Attitudes, and Knowledge about Breastfeeding

All 11 reviewed studies [51,52,53,54,55,56,57,58,59,60,61] examined mothers’ beliefs related to infant feeding. These studies found that most believed that “breastfeeding was natural” and “provided the best nutrition for infants’ health and growth”. Positive beliefs were found to be associated with breastfeeding initiation in some studies [51,54,57].

Moreover, four studies (4/11) found that mothers’ decision to breastfeed was partially influenced by her perceptions and personal assessment of infant’s health and growth [56,57,60,61]. For example, one qualitative study conducted in Canada [56] revealed that mother’s perceptions of their babies weight and size were used to assess whether her baby “was being fed high quality milk” and “enough milk”. Similarly, a qualitative study conducted in Australia [61] revealed that mothers’ who perceived their infants growth to be unsatisfactory (below 50%) were not confident that their milk supply was sufficient and that their infant was getting enough nutrition from breast milk alone.

In addition, despite studies indicating that women believed that breastfeeding is the best feeding mode, some studies reported that mothers often expressed doubts about being able to produce “enough breast milk” and perceived that breast milk alone would not satiate their baby, especially when infant’s growth “tracked down” [56,60,61]. A common belief among mothers was that a “fat baby is a healthy baby” [56,61]. This belief was associated with the decision to formula feed exclusively or in combination (mixed feeding) with breastfeeding [56,60,61].

Nine studies [51,52,53,54,55,56,57,60,61] assessed mothers’ attitudes towards breastfeeding, and overall these studies revealed that most mothers held favorable attitudes towards breastfeeding, and that most chose to breastfeed due to a sense of fulfillment, joy, love, and attachment to their baby. Nonetheless, several studies also reported common negative attitudes towards breastfeeding including breastfeeding being an embarrassing practice in pubic, adverse to mother’s figure, father feels left out, bottle feeding reduces risk of neonatal infection, breastfeeding babies become “too attached” to the mother [51,53,54,55,57,60,61]. In addition, four studies (4/9) found that mothers who held negative attitudes towards breastfeeding were less likely to breastfeed, and breastfed for shorter duration [54,57,60,61]. Moreover, some studies showed that mothers practiced mixed feeding (breast and formula feeding), and introduced formula in the first three months of infants’ life as they thought this would “familiarize their baby with formula” and prevent problems when breastfeeding was reduced or stopped [54,60,61].

Four studies assessed mothers’ knowledge of breastfeeding [51,53,55,57]. Overall, these studies revealed that Chinese immigrant mothers were aware of the health benefits of breast milk and advantages of breastfeeding their infants, although several misconceptions about breastfeeding existed [51,53,55,57]. A cross-sectional study conducted in Australia [53] found that more than half of the mothers (68.4%) believed in the importance of breast milk as the first feed for all infants and that infant formula should not be used as an initial food. Furthermore, a couple of studies (2/4) reported that mothers recognized colostrum as being nutritionally important and that the benefits of breast milk last after weaning [53,55]. Another cross-sectional study of Chinese mothers living in Australia found that although mothers were knowledgeable about the health benefits of breastfeeding, they also held several misconceptions such as bottle-feeding causes less neonatal infection, breastfed babies become “too attached” to the mother, and breastfeeding affects the healing of the episiotomy wound [51]. Similarly, a cross-sectional study conducted in Ireland [57] revealed that most Chinese immigrant mothers (approximately 82%) believed that breast milk is the ideal food for infants, over 70% were conscious of the unique health benefits of breast milk and more than 60% recognized that breastfeeding offered some disease protective effects. Nevertheless, some studies found that mothers held several misconceptions mothers held such as the benefits of breast milk last only until the baby is weaned, mothers should not breastfeed if have a cold, and infant formula should be fed to all newborns [51,56,57,61].

#### 3.2.3. Complementary Feeding (Introduction of Solids)

Five of the 11 included studies reported information on complementary feeding practices (i.e., introduction of solid foods) [54,56,57,60,61]. Finding of these studies conducted in three countries—Australia [54,61], Canada [56], Ireland [57], and the US [60] found that mothers introduced solid foods earlier than recommended. In addition, a cross-sectional study conducted in Australia [54] showed that Chinese immigrant mothers introduced solid foods earlier than Australian-born mothers. Two qualitative studies [60,61] showed that many of the infants’ solid foods introduced were traditional Chinese foods and that most were introduced before six months, most often at around three months of age. Mothers who introduced traditional Chinese solid foods to infants early did this as they perceived that that the benefits of this included strengthening bone development, children learning how to swallow foods other than milk, prolonged satiety, steady or accelerated growth, and improved digestive system based on the appearance of infants’ feces [60,61].

#### 3.2.4. Influence of Socio-Demographics, Economic and Cultural Factors on Infant Feeding

Nine of the 11 included studies examined the influence of socio-demographics, economic and acculturation factors on infant feeding beliefs and practices [51,52,53,54,55,56,57,60,61]. Two (2/9) cross-sectional studies conducted in Australia [53,54] found that higher family income was associated with mothers’ less preference for breastfeeding. Furthermore, two studies conducted in Australia [53,54] determined that mother’s level of education was positively associated with the initiation and increased duration of breastfeeding. Several studies documented that mothers from lower socio-economic and educational backgrounds more often reported misconceptions about breastfeeding and formula feeding than women with greater education and higher income [51,53,54,57]. Moreover, better employment opportunity for mothers in the country of immigration was found to be associated with shorter breastfeeding duration [54,57].

Cultural influences on infant feeding beliefs and practices were reported in several studies [51,56,57,60,61]. Four studies found that Chinese immigrant mothers’ breastfeeding practices (initiation and duration) are tied to traditional cultural health beliefs such as the *yin-yang* theory (hot-cold theory) and *zuo yuezi* (‘‘doing the month’’ or ‘‘sitting in for the first month’’) [56,57,60,61]. In addition to the belief about the consumption of “hot” or “warm” (e.g., protein rich) and “cold” (e.g., fruits and vegetables) foods during *zuo yuezi*, some studies also showed that mothers’ consumed specific protein-rich foods (e.g., fish, chicken, pork, duck, rabbit) to address breastfeeding issues such as low milk supply [56,57,60,61]. Moreover, one qualitative study [56] conducted in Canada found that mothers’ perception about breast milk was determined by their beliefs of physical health and restoring balance of the body.

Mothers’ level of acculturation to Western culture was found in both quantitative [52,54,57] and qualitative studies [56,60] to influence infant feeding practices. For example, some studies (5/11) found that mothers who had lived in the country of immigration longer were more likely to adopt infant feeding practices of that country, which in turn resulted in more positive infant feeding practices, including immediate initiation and longer duration of breastfeeding [52,54,60], and delayed introduction of solids foods [54,57].

#### 3.2.5. Family Support and Influence of Husbands and Grandparents on Infant Feeding Method

All 11 studies reported on the influence of family support on mothers’ infant feeding beliefs and practices, with studies documenting the critical influence of husbands and grandparents on mothers’ infant feeding beliefs and practices [51,52,53,54,55,56,57,58,59,60,61]. Three quantitative studies found that husband’s preference for breastfeeding was associated with breastfeeding initiation [53,54,55]. In addition, three other studies, two qualitative [60,61] and one quantitative [57] reported that grandparents in China often visit the mother’s new country and stay for several months to take care of the mother and the newborn. Two qualitative studies [60,61] showed that grandparents had a major influence on infant feeding decisions and practices, reporting that mothers often negotiated infant feeding choices, seeking agreement and support from extended family to align recommendations received from health professionals and extended family, in particular grandparents [60,61].

#### 3.2.6. Support from Healthcare Professionals

Four studies conducted in Australia reported on the influence of healthcare professionals on mothers’ infant feeding practices [53,54,55,61]. Three studies [53,54,55] using survey data from the same sample reported that Chinese women delivering in Australia received more breastfeeding support and assistance from health professionals and that these mothers were more likely to have breastfeed immediately after birth compared with women delivering in China. In addition, doctors’ support of breastfeeding had a positive influence on the initiation and duration of breastfeeding [53,54,55]. A qualitative study [61] revealed that mothers valued and respected the advice of health professionals such as doctors, nurses and midwives and relied on their advice to better understand the best infant feeding practices for healthy growth.

## 4. Discussion

Childhood obesity is a growing global public health epidemic [1,2]. The incidence of childhood obesity has increased in most high-income countries in the past decades, and more recently, the same trend is being observed in middle- and low-income countries [1,2]. Recent research documents increases in the prevalence of obesity in China [64,65,66] and among Chinese immigrants living outside China [67,68,69,70,71,72]. Despite some observed decreases in childhood obesity in high-income countries, racial/ethnic minority immigrant groups in these countries remain at increased risk [1,2,5,6,7,8,9,10,11,12]. Changing social and physical environments coupled with socioeconomic, language and cultural barriers, as well as limited access to, or inadequate utilization of healthcare represent additional risk factors for immigrant populations in a new country [27,73,74,75,76,77,78,79,80,81,82].

Maternal infant feeding practices are influential on infant’ and child’s growth, and could possibly explain obesity rates across generations [30,33,39,83]. A small, but increasing body of research conducted primarily in Western countries, has examined infant feeding practices of ethnic minority immigrant mothers [84,85,86,87], but limited attention has been paid to Chinese immigrant mothers, a large and growing group in several countries. Therefore, the purpose of this integrative review was to identify and synthesize information from studies examining infant feeding practices conducted among Chinese immigrant mothers in order to describe and highlight factors influencing infant feeding practices of this immigrant group that may be used to inform the design of interventions.

Overall, findings of studies included in this integrative review showed high initiation rates of breastfeeding, but sharp decline in rates of breastfeeding by six months of age [51,52,53,54,55,58,59]. The limited duration of EBF is concerning, as a longer duration is associated with positive health outcomes for infant and mother [19,27,30,33,34,38,40].

Findings of studies included in this review showed that overall Chinese immigrant mothers hold positive beliefs about breastfeeding [51,53,54,55,56,58,59,60,61]. Nonetheless, reviewed studies also documented negative beliefs and misconceptions such as bottle feeding causes less neonatal infections, breastfed babies become “too attached” to the mother, etc. [51,52,53,55,56,58,60] that may influence mothers’ decision to supplement infant feeding with formula. Furthermore, several studies found that most Chinese immigrant mothers do not EBF, and that the concomitantly use of formula feeding is common [53,55,58,60,61]. These findings are in agreement with findings from studies conducted in China [88,89,90,91]. Interventions designed to promote healthy infant feeding practices among Chinese immigrant mothers and families should take this information into account. Moreover, obesity prevention and control efforts among Chinese children of immigrant families should target early feeding practices including practices during infancy, as these practices may be closely related to the early origins of obesity risk in this population group.

Cultural, socioeconomic, and psychological factors shape mothers’ perceptions of and practices related to infant feeding [27,30,33]. Cultural dietary and feeding beliefs are particularly important for immigrant populations, which often hold on to these cultural beliefs across generations to keep a close relationship with their country and cultural origins [88,89,90,91]. In agreement with prior research conducted in a range of cultural settings [92,93], findings from studies included in this review documented the influence of culture beliefs on mothers’ infant feeding practices [51,53,54,55,56,57,58,59,60,61]. Evidence shows that many traditional medical practitioners in China encourage exclusive breastfeeding during the practice of *zuo yuezi* for the benefit of both mother and infant [88,89,90,91]. These findings suggest the importance of understanding and incorporating cultural beliefs common in the Chinese culture when developing interventions to promote healthy infant feeding practices targeting Chinese immigrant mothers and families. Moreover, findings from a few reviewed studies suggest the influence of acculturation levels on Chinese immigrant mothers’ infant feeding beliefs and practices [54,56,58,59,60,61], and indicate the importance of taking into account acculturation levels. Interventions developed to promote healthy infant feeding practices targeting Chinese immigrant mothers should be designed taking social and cultural changes faced by families and mothers, as well as acculturation levels within Chinese immigrant families.

Like previous studies with other ethnic groups, findings from studies included in this review indicate that mothers’ infant feeding beliefs and practices are influenced by the broader family context [93,94,95,96]. Findings from studies reviewed showed that husbands and grandparents are important influences on Chinese immigrant mothers’ infant feeding beliefs and practices, which is in agreement with previous research conducted in China suggesting that family roles in Chinese culture influence mother’s choice to breastfeed [97,98,99,100]. Studies conducted in China indicate that in Chinese culture, the baby’s father and paternal grandmother have significant influence on the choice of infant feeding method [88,89,90,98,99]. In the case of Chinese immigrant mothers, the husband might be particular influential on the decision to breastfeed [91]. These findings are important and suggest that effective interventions targeting Chinese immigrant mothers must address the promotion of healthy infant feeding practices within the context of the family, and take into account the influence of family members, especially husbands and grandparents, who should also be considered targets for health promotion interventions.

Although findings from studies included in this review documented the influence of socio-demographic and economic factors on Chinese immigrant mothers’ infant feeding practices [51,52,53,54,55,56,58,59,60,61], the mothers participating in the majority of the studies (8/11) were from relatively affluent backgrounds. Overall, the majority of Chinese immigrant mothers participating in the majority of the reviewed studies conducted in Australia (7/11) [51,52,53,54,55,58,59,61] and Ireland (1/11) [57] were highly educated (50%–80% participants had completed college), and most were relatively affluent (middle income or higher). Mothers’ ages ranged from 22 to 59 years old, the majority had resided in the country of immigration for more than five years, and most were housewives. Nonetheless, in agreement with research conducted among other ethnic minority groups [27,33,42,74,79,82,87,98], findings from reviewed studies suggest the importance of taking into account socio-demographic and economic factors when designing interventions for Chinese immigrant children and families.

Mirroring findings from research conducted with other ethnic groups [43,83,84,85,86,87,94], findings from studies included in this review showed that early introduction of solids is a common practice [54,56,57,60,61], and that cultural beliefs might influence this practice [57,60,61]. Furthermore, a few of reviewed studies showed that some mothers were not knowledgeable about the long-term benefits of breastfeeding, which may result in the early introduction of solids [54,56,57]. Conflicting evidence exists for the association between timing of introduction of solid foods and risk of subsequent obesity [34,42,94,101,102,103]. Some studies suggest that early introduction of solid foods is associated with subsequent obesity in childhood, and that the association vary by whether an infant is breastfed or formula-fed [34,42,101,102]. While some studies have found that breastfeeding and delaying complementary foods yield lower likelihood of obesity and greater probability of healthy weight status [19,34,38,42], other studies have not found a significant association between early introduction of solids and risk of obesity in childhood [43].

Our evaluation of the methodologies of studies included in this integrative review suggests some possible limitations, which warrant caution in the interpretation of study findings. All included quantitative studies (8/8) examining infant feeding practices were cross-sectional. Additional longitudinal studies are needed to better understand infant feeding practices, and the relationship between these practices and risk of overweight and/or obesity in children in Chinese immigrant families. All included quantitative studies employed self-reported or administered questionnaires for the assessment of infant feeding practices, which may result in misclassification. A methodological strength was the use of well-known, validated questionnaires to assess infant feeding knowledge, attitudes and practices including the PIFS [62] and the IIFAS [63] in six of the reviewed studies [53,54,55,57,58,59].

Only three qualitative studies were identified and included in this review [56,60,61], and these studies were conducted in three countries including Australia [61], Canada [56], and the U.S. [60]. Given the dearth of information on Chinese immigrant mothers’ infant feeding practices, additional qualitative studies are needed to further explore factors identified by available qualitative and quantitative studies conducted among this population group.

Finally, the majority (8/11) studies examining infant feeding practices were conducted among Chinese immigrants in Australia [51,52,53,54,55,58,59,60,61], with only three studies conducted in three other countries (i.e., Canada, Ireland, and the U.S.) [56,57,60]. Although several countries with large Chinese immigrant populations (e.g., Australia, Canada, Ireland, and U.S.) were represented in studies included in this review, other countries with large Chinese immigrant populations (e.g., Thailand, Singapore, Malaysia, Indonesia, Europe) were not. It is probable that Chinese immigrants in countries not included in this review are influenced by similar factors that Chinese immigrants in countries represented in this review, even though some social contextual factors may differ. Furthermore, the paucity of studies examining infant feeding practices of immigrant Chinese mothers in countries with large Chinese immigrant populations such as Canada and the U.S. is a concern given the evidence from research linking infant feeding to increased risk of child obesity during early childhood [19,34,35,36,37,38,39,40], and evidence that children of racial/ethnic minority families living in high-income countries are at increased risk of obesity [5,6,7,8,9,10,11,12]. Finally, it is possible that we did not identify all relevant articles due to studies being published in other formats, such as country reports, alternative databases, or in other languages not included in this review. The use of systematic criteria (i.e., PRISMA) [47] to identify and select studies and modified quality assessment tools for the critical appraisal of papers are strengths of this study (i.e., STROBE and CASP) [49,50].

In summary, additional studies are needed to further explore and understand multiple influences on Chinese immigrant mothers’ infant feeding decisions and practices and to quantify the effects of these practices on child’s weight status in infancy and early childhood. Future research should also include both qualitative and quantitative research methods and longitudinal study designs and further explore the role of psychosocial and cultural factors in influencing infant feeding practices of Chinese immigrant parents. In addition, studies employing multiple methods for assessing infant feeding practices including direct observations are needed to further examine infant feeding practices associated with increased risk of overweight and obesity in children of Chinese immigrant families. This information will be important to identify factors among this ethnic group amenable to intervention.

## 5. Conclusions

Information about factors associated with parental feeding practices is needed to promote healthy infant feeding practices aimed at the prevention of child overweight and obesity [103]. Several modifiable infant feeding beliefs and practices were identified in the present integrative review. Findings from the synthesis of studies included in this integrative review suggest direction for further research, as well as potential targets for interventions aimed at promoting healthy infant feeding practices and preventing and decreasing disparities in early childhood obesity among Chinese children of immigrant families. Given the growing evidence suggesting the link between infant feeding practices and increased risk of overweight and obesity in children, additional research examining these associations in Chinese immigrant children in various geographical location (i.e., Asia, Australia, Europe and North America) is needed due to the paucity of research identified and the increasing prevalence of overweight and obesity rates in Chinese children of immigrant families in several countries across the globe. This information is needed to develop culturally relevant interventions that can change influences in these modifiable risky practices and that are likely to help prevent and reduce risk of child obesity in Chinese children of immigrant families.

## Supplementary Materials

The following are available online at www.mdpi.com/1660-4601/15/1/21/s1, Figure S1: PRISMA flow diagram, Table S1: Quality assessment of included quantitative studies using adapted “Strengthening the Reporting of Observational Studies in Epidemiology (STROBE)” statement; Table S2: Quality assessment of included qualitative studies using the Critical Appraisal Skills Program (CASP); Table S3: Description of studies included in systematic review; Table S4: Characteristics of studies examining infant feeding beliefs, attitudes, knowledge and practices of Chinese immigrant mothers included in integrative review.
