# Supplementary material for: Infant Feeding Beliefs, Attitudes, Knowledge and Practices of Chinese Immigrant Mothers: An Integrative Review of the Literature
Source: Int J Environ Res Public Health. 2017 Dec 23;15(1):21. doi: 10.3390/ijerph15010021 (PMC5800121; doi:10.3390/ijerph15010021)
Supplement: Supplementary file 1 [file ijerph-15-00021-s001.pdf]

Figure S1: PRISMA 2009 flow diagram*

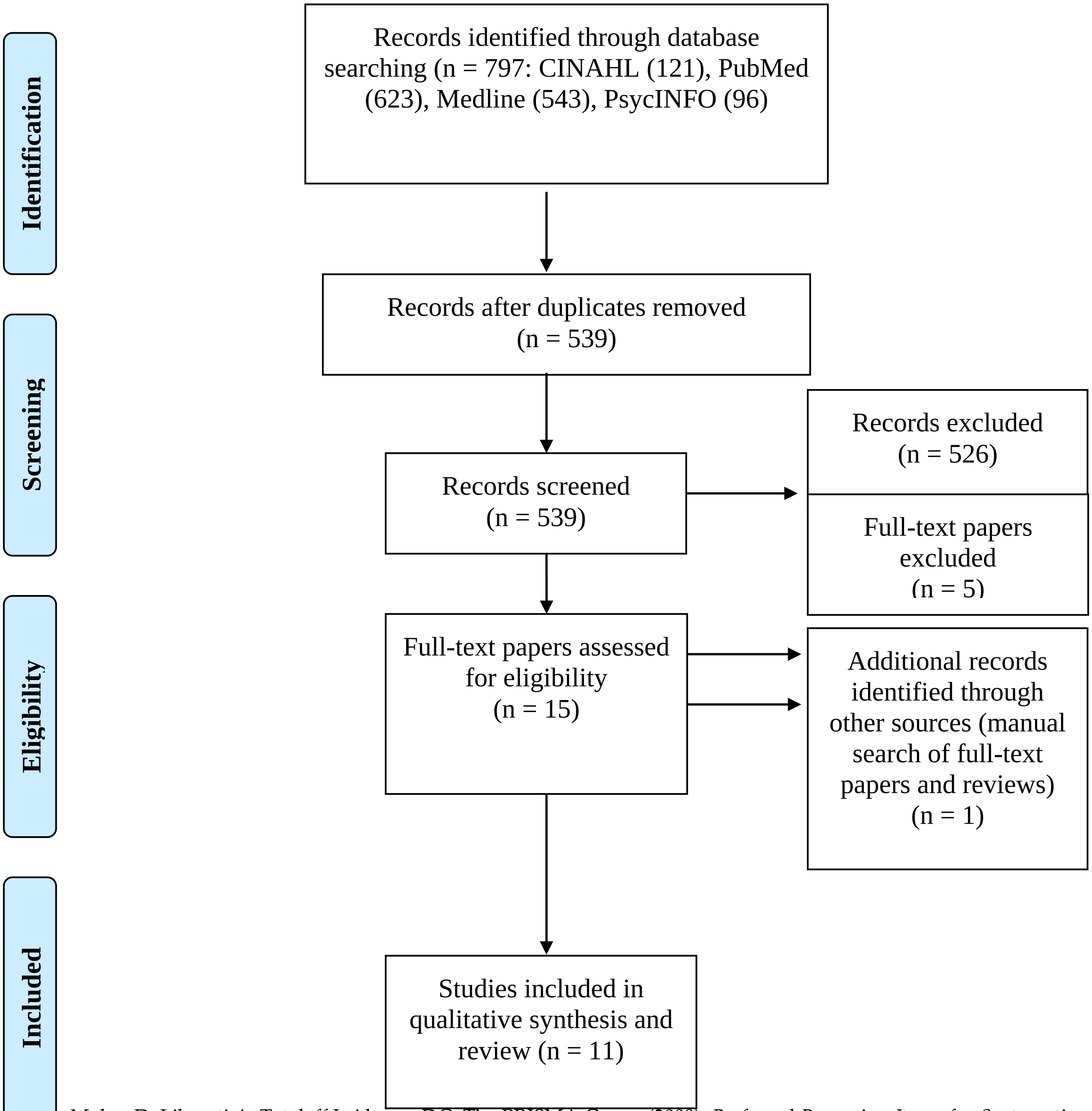

From: Moher D, Liberati A, Tetzlaff J, Altman DG, The PRISMA Group (2009). *P*referred *R*eporting *I*tems for *S*ystematic Reviews and *M*eta-*A*nalyses: The PRISMA Statement. PLoS Med 6(7): e1000097. doi:10.1371/journal.pmed1000097

Table S1. Quality assessment of included quantitative studies using adapted "Strengthening the Reporting of Observational Studies in Epidemiology (STROBE)" statement (n=8)

Items:

#1. Is the study longitudinal?
#2. Does the paper describe the participants' eligibility criteria?
#3. Were study participants randomly selected (or representative of the study population)?
#4. Did the paper report information about the measures, including references used to assess parental feeding practices?
#5. Did the study include information on instrument or scale used to assess parental feeding practices that have acceptable reliability?
#6. Did the study provide information power calculation to detect hypothesized relationships?
#7. Did the study report the number of individuals who completed each of the different measures?
#8. Did the participants/respondents complete at least 80% of measures?
#9. Did analyses take into account confounding factors?

	Items									
**Studies**	**#1**	**#2**	**#3**	**#4**	**#5**	**#6**	**#7**	**#8**	**#9**	**Total**
Diong et al. [51]	0	1	0	1	1	0	0	0	1	4
Li et al. [53]	0	1	1	1	0	0	0	1	1	5
Li et al. [54]	0	1	1	1	0	0	0	1	1	5
Li et al. [55]	0	1	1	1	0	0	0	1	1	5
Zhou et al. [57]	0	1	0	1	1	0	1	1	1	6
Chen et al. [58]	0	1	1	1	1	0	0	1	1	6
Chen et al. [59]	0	1	1	1	1	0	0	1	1	6
Homer et al. [52]	0	1	1	1	1	1	0	0	1	7

Table S2. Methodological assessment of qualitative studies included in integrative review (n=3).

**Qualitative Evaluation Criteria**	Lee et al. [60]	Kuswara et al. [61]	Chen W. [56]
Was there a clear statement of the aims of the research?	Y	Y	Y
Was the research design appropriate to address the aims of the research?	Y	Y	Y
Was the recruitment strategy appropriate to the aims of the research? (e.g., How were participants selected? e.g. purposive, convenience, consecutive, snowball?; How were participants approached? e.g. face-to-face, telephone, mail, email?)	Y	Y	Y
Were data collected in a way appropriate to address the research questions? (e.g., Were questions, prompts, guides provided by the authors? Was it pilot tested? Was data saturation discussed?)	Y	Y	Y
Has the relationship between researcher and participants been considered?	N	N	N
Have ethical issues been taken into consideration?	Y	Y	Y
Was the data analysis sufficiently rigorous?	U	Y	Y
Is there a clear statement of findings?	Y	Y	Y
Is the research valuable?	Y	Y	Y

Y=yes, N=no, U=uncertain.

Table S3. Description of studies included in integrative review (n=11)

Characteristics	No. of Studies
**Total number of studies selected**	11
**Publication dates**	
2000 – 2005	5
2006 – 2011	2
2012 – 2017	4
**Research Methods (study design)**	
Qualitative	3
Quantitative (cross-sectional)	8
**Countries represented**	
United States	1
Australia	8
Canada	1
Ireland	1
**Feeding practices**	
Infant feeding	11
Breastfeeding	7
Breastfeeding and complementary feeding	4
**Assessment of parental infant and child feeding practices**	
Semi-structured interviews	3
Iowa Infant Feeding Attitude Scale (IIFAS) [62]	2
Perth Infant Feeding Study (PIFS) questionnaire [63]	3
Other (i.e., medical records, questionnaires developed specifically for study)	3

Table S4. Characteristics and findings of studies examining infant feeding beliefs, attitudes, knowledge and practices of Chinese-immigrant mothers living outside China included in integrative review (n = 11).

Author (Year) Country	Sample characteristics and study design	Study Aim (s)	Measures of infant and/or child feeding practices	Main Findings
**Qualitative Studies**				
Lee et al. [60] United States	n = 22 (immigrant mothers) Mothers' age range: 24 – 35 years. Infants' age range: 0 – 12 months. Qualitative study including semi-structured individual interviews.	To examine the influence of elders and cultural beliefs on postpartum, infant feeding, and childcare practices.	Semi structured interviews with mothers using interview guide.	- The traditional postpartum practice, *"zuo yuezi"* influenced maternal delay in lactation.<br>- Early introduction of solids for traditional reasons was reported.<br>- Mothers' believed that the support from husbands and elders was necessary for breastfeeding success. Some mothers had to first negotiate with elders for breastfeeding.
Kuswara et al. [61] Australia	n = 36 (immigrant mothers)<br><br>Mothers' age range: 21–40 years. Infants' age range: 0 – 12 months<br><br>Qualitative study including semi-structured individual interviews.	To explore Chinese immigrant mother's experiences of feeding their infant to gain an insight into the factors shaping their feeding decisions and perceptions of infant growth.	Semi structured interviews with mothers using interview guide.	- Mothers were supportive of exclusive breastfeeding, however breastfeeding problems and conflicting views about infant feeding and infant growth from grandparents reduced many mothers' confidence to breastfeed exclusively.<br>- For many new mothers, anxiety that exclusive breastfeeding provided insufficient nourishment led to the introduction of formula before six months of age.<br>- Most mothers delayed introducing solid food to five to six months to prevent development of allergic diseases and gastrointestinal problems.<br>- Mothers obtained information and support related to infant feeding from a combination of health professionals, online resources, friends and grandparents.
Chen W. [56] Canada	n = 15 (immigrant mothers)<br><br>Mothers' age range: 21–41 years. Infants' age range: 0 – 2 months.<br><br>Qualitative study including semi-structured individual interviews.	To explore Chinese mothers' perceptions about breastfeeding and infant health in the Canadian context.	Semi structured interviews with mothers using interview guide.	- Two main themes emerged: 1) the idea of harmony within change and 2) the meaning of infant health.<br>- The first represented mothers' perceptions about breastfeeding: the value of common sense, purity of breast milk and the laws of nature. The second represented the notions of infant health, including its indicators and the relationship between mother's health and infant health.<br>- Chinese immigrant mothers' concepts of breastfeeding were associated with Western biomedical thought, traditional Chinese medicine and personal experiences, especially those embedded in the traditional Chinese cultural context.<br>- Perceptions of breastfeeding and infant health regarding notions of harmony within natural dynamic patterns must be considered when promoting breastfeeding.

Author (Year) Country	Sample characteristics and study design	Study Aim (s)	Measures of infant and/or child feeding practices	Main Findings
**Quantitative Studies**				
Li et al. [55] Australia	n = 506 (immigrant mothers)<br><br>Mothers' age range: 23–59 years.	To explore differences of infant feeding practices in the mothers' home countries and Australia for Mandarin-speaking women who had children born in Australian or overseas, and were living in Perth, Western Australia at the time of the study.	PIFS questionnaire (telephone interview) containing items related to knowledge and attitudes towards breastfeeding with additional questions specific for Chinese culture.	There were no significant differences between any breastfeeding rates among Chinese women delivering in their home countries where breastfeeding initiation was 86.6% or in Australia.<br><br>Full breastfeeding rates at three and six months for Chinese immigrant were lower than rates for Australian mothers.<br><br>Chinese mothers delivering in Australia received more breastfeeding support and assistance from health professionals that Chinese mothers delivering in their home countries.<br><br>In Australia, 78% stated that they received support form their doctors and nurses for breastfeeding compared with 37.6% in their home countries ($P < 0.001$). In particular, they were more likely to have put their infant to the breast immediately after birth (47.9%) compared with women delivering in their home countries (4.1%) ($P < 0.001$).<br><br>Solid foods were introduced earlier to infants of Chinese mothers living in their home countries.<br><br>The Chinese immigrant mothers' infant feeding practices in Australia reflected both Western and Eastern influences. Chinese immigrant mothers introduced complementary foods earlier than in other Australian studies, suggesting that culturally specific programs will be needed to encourage exclusive breastfeeding to six months.
Chen et al. [59] Australia	n = 200 (Chinese immigrant mothers living in Perth and 1620 mothers living in Chengdu, China).<br><br>Mothers' (living in Perth, Australia) mean age: 33.5±5.0 years.	To compare the infant feeding attitudes of Chinese mothers living in Perth Australia and in Chengdu, China.	The IIFAS	The mean IIFAS scores in both country groups lay in the range of 'neutral breastfeeding attitudes'. Higher IIFAS scores were significantly associated with the likelihood of both breastfeeding initiation (OR: 3.85; CI: 2.49-5.96; $p<0.001$) and longer (≥8 months) breastfeeding duration (OR: 2.52; CI: 1.87-3.40; $p<0.001$).<br><br>Chinese mothers in Perth (mean IIFAS score=60.0±6.3) tended to have more positive attitudes towards breastfeeding than mothers in Chengdu (mean IIFAS score=57.7±5.1, $p<0.001$), and had a longer duration of 'any breastfeeding' (10.0±6.2 months in Perth compared to 7.4±4.3 months in Chengdu, $p<0.001$).

Author (Year) Country	Sample characteristics and study design	Study Aim (s)	Measures of infant and/or child feeding practices	Main Findings
**Quantitative Studies (cont.)**				
Chen et al. [58] Australia	n = 239 (Chinese immigrant mothers living in Perth and 1844 mothers living in Chengdu, Sichuan Province, PR China).	To compare the initiation and duration of breastfeeding between Chinese Australian migrants and Chinese mothers in mainland China and test the healthy migrant effect in Chinese Australian migrants in Perth, Western Australia.	The IIFAS	Breastfeeding initiation rate in Chinese immigrant mothers (94.1%) living in Australia was higher than in mothers in China (86.2%, P < .001). Chinese immigrant mothers also had longer breastfeeding duration, greater "full breastfeeding" rate at 6 months, and greater "any breastfeeding" rates at 6 and 12 months.<br><br>After controlling for potential confounding variables, the results of the binary logistic regression analysis showed that the location of the mother (Australia or China) was associated with breastfeeding practices.<br><br>Chinese mothers living in Chengdu were less likely to initiate breastfeeding (odds ratio = 0.47, 95% confidence interval, 0.25-0.89) and breastfeed their babies at 12 months (odds ratio = 0.48, 95% confidence interval, 0.33-0.69) than Chinese mothers in Perth. The higher breastfeeding initiation and longer breastfeeding duration by Chinese migrant mothers in Perth than mothers in Chengdu suggest a "healthy migrant effect."
Li et al. [54] Australia	n = 506 (immigrant mothers)<br><br>Mothers' age range: 23–59 years.	To identify determinants of the initiation and duration of breastfeeding by Chinese Australian mothers.	PIFS questionnaire (telephone interview) containing items related to knowledge and attitudes towards breastfeeding with additional questions specific for Chinese culture.	Doctors' support of breastfeeding was positively associated with the initiation of breastfeeding both in the mothers' home countries and in Australia and with duration.<br><br>Mother's level of education was positively associated with the initiation of breastfeeding in the mother's home country (OR, 3.04; 95% CI, 1.36-6.80) and positively associated with the duration of breastfeeding both in the mother's home country and Australia.<br><br>Father's preference for breastfeeding was positively associated with the initiation of breastfeeding of the mother giving birth in Australia (OR, 4.96; 95% CI, 1.93-12.66).<br><br>Health professionals can provide invaluable support for mothers initiating and continuing breastfeeding in this population. Prenatal education also needs to emphasize the ways in which fathers can support and contribute to breastfeeding.

Author (Year) Country	Sample characteristics and study design	Study Aim (s)	Measures of infant and/or child feeding practices	Main Findings
**Quantitative Studies (cont.)**				
Diong et al. [51] Australia	n = 101 (immigrant mothers)<br><br>Mothers' age: not reported.	To investigate the breastfeeding rates of immigrant Chinese mothers.<br>To examine factors that influence breastfeeding and bottle-feeding in this population, as well as the knowledge or misconceptions of these Chinese mothers had about breastfeeding.	Questionnaire devised for the present study based on review of literature.	Sixty-five percent of Chinese immigrant mothers were fully breastfeeding their infants at hospital discharge, with a further 6.9% partially breastfeeding. However, only 34% were still breastfeeding at three months.<br><br>The most important factor influencing mothers' choice to breastfeed was the belief that it was 'good for the baby', while mothers choosing to bottle-feed were influenced by the belief of "low milk supply", and the belief that bottle feeding was easier.<br><br>Similar beliefs about breastfeeding and bottle-feeding were held by Australian and Chinese immigrant mothers, although concerns about the baby becoming too attached were markedly higher in the immigrant group and may be related to sudden drop in breastfeeding rates at three months.<br><br>Findings suggest the need to focus on ethno-specific services including creating a support network for Chinese immigrant mothers to promote longer breastfeeding duration up to six months of the infant's life.
Li et al. [53] Australia	n = 506 (immigrant mothers)<br><br>Mothers' age range: 23–59 years.	To describe Chinese-Australian (Mandarin speaking) mothers' knowledge about and attitudes towards breastfeeding.	PIFS questionnaire (telephone interview) containing items related to knowledge and attitudes towards breastfeeding with additional questions specific for Chinese culture.	Most mothers (90.9%) indicated that they supported breastfeeding for all infants and most had some knowledge about the benefits of breastfeeding.<br><br>The main reasons mothers considered stopping breastfeeding were "not having enough breast milk" or going back to work or study.<br><br>The higher the family income, the less preference toward breastfeeding.<br><br>Findings highlighted the significance of social and cultural factors that impact women's to initiate and maintain breastfeeding.

Author (Year) Country	Sample characteristics and study design	Study Aim (s)	Measures of infant and/or child feeding practices	Main Findings
**Quantitative Studies (cont.)**				
Zhou et al. [57] Ireland	n = 322 (immigrant mothers)<br><br>Mothers' age range: 22 to 54 years old (mean age 33.2 years).<br><br>Cross-sectional	To explore knowledge and attitudes related to breastfeeding of Chinese mothers living in Ireland.	Questionnaire devised on the basis of an extensive literature review of migration studies.	In spite of considerable awareness of the advantages of breastfeeding (mean score = 4.03 ± 0.73), some misconceptions (e.g. 'mother should stop breastfeeding if she catches a cold') and negative attitudes (e.g. breastfeeding inconvenient, embarrassing, and adverse to mothers' figure) existed, especially among the less educated mothers.<br><br>Cultural beliefs concerning the traditional Chinese postpartum diet were prevalent, particularly among those who had lived in Ireland for a shorter duration ($P = 0.004$).<br><br>Psychological parameters had strong independent associations with breastfeeding practices in this study. Those who had lower awareness score (OR = 2.98, 95% CI: 1.87-4.73), more misconceptions and negative attitudes ($P < 0.05$), and weaker cultural beliefs ($P < 0.05$) were less likely to breastfeed.
Homer et al. [52] Australia	n = 180 (Chinese immigrant mothers; the total study sample also included 160 Arabic-speaking and Australian/English-speaking mothers).<br><br>Mothers' mean age: 32 (SD = 4) years	To examine early infant feeding decisions and duration of breastfeeding in 986 women from English, Chinese, and Arabic-speaking backgrounds in Sydney during 1997 and 1998.	Medical records and questionnaire (8 weeks postpartum) devised for the study.	Chinese-speaking women were less likely to express an intention to breastfeed and fewer initiated breastfeeding compared with other women. A greater proportion of the Chinese-speaking women who initiated breastfeeding were still breastfeeding at 8 weeks compared with English-speaking women.
